# Should checkpoint inhibitors be reserved for biomarker-selected pediatric brain tumors?

**DOI:** 10.1007/s11060-026-05605-4

**Published:** 2026-05-16

**Authors:** Yaxel Levin-Carrion, Jayant Bhasin, Kevin Titkov, Sraavya Anne, Arman Sawhney, Caryn J. Ha, Ibraheem Sharaf, Marvens Jean, Jacob Santana, Drew Thibault, Alejandro Pando, Nemanja Novakovic, Nehal S. Parikh, Morana Vojnic, Jonathan H. Sherman

**Affiliations:** 1 Rutgers New Jersey Medical School, Newark, NJ USA; 2 Department of Neurological Surgery, Rutgers New Jersey Medical School, Newark, NJ USA; 3 Rutgers Biomedical and Health Sciences, Newark, NJ USA; 4 Department of Neurological Surgery, Corewell Health William Beaumont University Hospital, Royal Oak, MI USA; 5 Department of Pediatric Hematology and Oncology, Rutgers Robert Wood Johnson Medical School, New Brunswick, NJ USA; 6 Department of Neuro Oncology, Rutgers Cancer Institute of New Jersey, New Brunswick, NJ USA; 7 Department of Neurosurgical Oncology, Rutgers Cancer Institute of New Jersey, New Brunswick, NJ USA

**Keywords:** Immune checkpoint inhibitors, PD-1/PD-L1 blockade, CTLA-4, Hypermutation, Mismatch repair deficiency, Tumor immune microenvironment, Pediatric CNS tumors

## Abstract

**Purpose:**

Pediatric brain tumors, including high-grade gliomas (HHG), medulloblastomas (MB), and ependymomas (EPN), are a leading cause of death in children. They are often immunologically “cold” with low tumor mutational burden (TMB) and very few tumor-infiltrating lymphocytes (TILs), which may limit the role of immune checkpoint inhibitors (ICI).

**Methods:**

We performed a PRISMA‑guided systematic review of PubMed/MEDLINE, Embase, and Scopus from inception to September 17, 2025, for English-language studies of patients ≤ 21 years with primary CNS tumors treated with PD‑1/PD‑L1 or CTLA‑4 inhibitors. Eligible reports included prospective trials, retrospective series, and observational/case reports with extractable data on efficacy and/or toxicity. Of 479 records identified, 386 unique citations were screened, 127 underwent full‑text review, and 40 met inclusion criteria for qualitative synthesis.

**Results:**

Prospective and institutional studies in biomarker-unselected diffuse midline glioma, high-grade glioma, medulloblastoma, and ependymoma showed low objective response rates (generally ≤ 6%), short median progression-free survival (1–3 months), and overall survival similar to historical controls, despite acceptable safety (grade ≥ 3 treatment-related adverse events ~ 15–25% with anti-PD-1 ± anti-CTLA-4). In contrast, across germline MMR-deficient and broader RRD/hypermutant cohorts, PD-1 blockade produced clinically meaningful and sometimes durable responses, including complete remissions in malignant glioma and approximate 2-year overall survival near 50%, often with delayed responses and pseudoprogression. Histology-specific profiling highlighted marked variation in immune contexture: pediatric gliomas segregate into immune “hot,” “altered,” and “cold” subtypes; medulloblastoma is largely PD-L1–low with prominent B7-H3 and myeloid programs; checkpoint expression is also observed in germ cell and selected sellar tumors. Evidence quality is limited by small, heterogeneous, predominantly non-comparative designs.

**Conclusion:**

ICIs show manageable safety but limited efficacy in unselected pediatric CNS tumors. Durable benefit is most evident in RRD/hypermutant biology and possibly PD-L1–high niches (e.g., some low-grade gliomas and CNS-GCT). Future trials should be biomarker-driven and pair ICIs with priming combinations (e.g., radiation, epigenetic modulators, metronomic chemotherapy) to convert “cold” tumors into responders.

**Supplementary Information:**

The online version contains supplementary material available at 10.1007/s11060-026-05605-4.

## Introduction

Pediatric CNS tumors are now the leading cause of cancer-related mortality in children, emphasizing the need for novel and effective therapeutic approaches. Checkpoint blockade targeting PD-1/PD-L1 or CTLA-4 unleashes anti-tumor T-cell responses and has reshaped the management of adult melanoma, lung, renal, and other cancers. However, translation to both adult and pediatric CNS tumors has achieved only limited success and has been challenging for mechanistic and clinical reasons: lower average tumor mutation burden (TMB), distinct epigenetics and antigen landscapes, inhibitory myeloid immune cell recruitment, and the tight anatomical and immunological regulation of CNS immune responses [1–5]. However, compelling exceptions exist, especially in the replication/mismatch repair-defective childhood cancers (RRD/CMMRD) where hypermutation may generate sufficient neoantigens for effective immune recognition [6–9]. Emerging research demonstrates that the pediatric brain tumor immune microenvironment (iTME) is neither uniformly “cold” nor devoid of antitumor potential. Single-cell profiling of pediatric brain tumors shows clonally expanded CD8 and CD4 T cells with effector features and checkpoint expression, and neoantigen-specific T-cell gene signatures track with better outcomes [10]. Conversely, comprehensive immune-contexture analyses across pediatric CNS tumor confirm that medulloblastoma is often characterized by a lack of immune activity and has low levels of PD-L1, which helps explain the lack of immune checkpoint inhibition (ICI) monotherapy success in unselected cohorts [11–13]. Replication-repair–deficient (RRD) brain tumors in children are uniformly hypermutant or MSI-high and show dense T-cell infiltration, providing a biologic rationale for checkpoint blockade, and recent reviews emphasize pursuing combinations after anti-PD-1 failure alongside noninvasive monitoring [14]. Early pediatric combination strategies are also emerging; for example, a safety run-in evaluating nivolumab layered onto metronomic chemotherapy established feasibility in children and adolescents, supporting trials that ‘prime’ the immune milieu [15].

Here we report a systematic review of all studies examining ICIs in pediatric CNS tumors. Given the rarity and biological heterogeneity of these tumors, along with the logistical challenges of conducting prospective immunotherapy studies in children, available evidence has remained fragmented and difficult to interpret in aggregate. We systematically searched the literature for reports describing ICI use in patients ≤ 21 years of age with primary CNS tumors, including prospective interventional studies, retrospective series, and observational reports that detailed clinical response, survival, or immune-related adverse events. Our primary aim was to characterize efficacy signals and safety profiles across tumor histologies and molecular subgroups. Secondary objectives included cataloging biomarkers of response, patterns of progression, and emerging combination strategies. This review consolidates current clinical data to establish the scope and utility of ICI in pediatric brain tumors and further establish how future trials can best continue to explore their potential.

## Methods

### Protocol

We conducted and reported this systematic review in accordance with PRISMA 2020 guidance. An a priori protocol specified objectives, eligibility criteria, outcomes of interest, data-management plans, and the analysis plans. Because this review synthesized published data only, de-identified data exclusively, requirements for institutional review board approval and patient consent did not apply.

### Data sources and search strategy

We searched PubMed/MEDLINE, Embase, and Scopus from database inception to September 17, 2025, limited to English-language records. The search strategy combined controlled vocabulary (MeSH/Emtree) and free-text synonyms across three concepts: pediatric populations; primary central nervous system tumors (histology-specific terms included glioma/DIPG/DMG, glioblastoma, medulloblastoma, ependymoma, pineoblastoma, ATRT/ETMR, and craniopharyngioma); and immune checkpoint inhibition (PD-1/PD-L1, CTLA-4, and agent names). We used truncation and American/British spellings, mapped terms to title/abstract fields where appropriate, and an English-language limit was applied across databases. No date or study-design filters were used. The full, reproducible strategies for each database (with field tags, Boolean logic, and limits) and per-database results counts are provided in Supplementary eMethods Table S1. A total of 479 records were identified through our comprehensive database search, including 264 from PubMed, 151 from Scopus, and 64 from Embase. After automated and manual duplicate removal, 93 duplicative records were excluded, leaving 386 unique titles and abstracts for initial screening. Title/abstract and full-text screening were performed independently by two reviewers using Covidence, with discrepancies resolved by consensus.

### Eligibility criteria and study selection

During this stage, studies were excluded for reasons such as non–CNS primary tumor type, adult-only patients populations, use of intervention other than immune-checkpoint inhibitor, lack of extractable clinical outcomes, or an exclusive focus on preclinical/animal model work. After systematically reviewing titles and abstracts, 259 records did not meet the inclusion criteria and were removed. This screening step refined the pool to studies more directly relevant to clinical decision making in pediatric CNS tumors, ensuring that subsequent analyses drew from evidence with the greatest potential to inform real world therapeutic understanding.

### Data extraction and synthesis

The remaining 127 studies underwent detailed full-text assessment to confirm eligibility according to predefined criteria. These criteria include patient age (≤ 21 years), a clearly documented primary CNS tumor diagnosis, exposure to an immune-checkpoint inhibitor, and reporting of clinical efficacy and/or toxicity outcomes in a manner that could be reliably extracted. Studies were excluded when clinical data were insufficient for abstraction, when pediatric cases were embedded within mixed age cohorts without stratified reporting or when manuscripts focused exclusively on laboratory or imaging biomarkers without linking findings to treated patients. Through this process, 40 studies met all criteria and were included in the final qualitative synthesis. The resulting cohort reflects the most complete clinically oriented evidence available for pediatric CNS tumors treated with immune checkpoint inhibitors, providing a concentrated foundation for interpreting therapeutic patterns, safety and early indications of activity in this understudied population. Data items extracted included tumor histology and molecular subgroup; clinical setting (newly diagnosed vs. recurrent/progressive); patient age; immune checkpoint inhibitor (ICI) agent(s) and dose/schedule when available; prior and concomitant therapies (surgery, radiotherapy, systemic chemotherapy/targeted therapy) and corticosteroid exposure when reported; objective response and disease control endpoints; progression-free survival (PFS) and overall survival (OS); and immune-related adverse events.

Biomarker definitions: Because PD-L1 and tumor mutational burden (TMB) testing is not standardized across pediatric CNS tumor studies, we recorded the assay type and the positivity/high thresholds used in each report. In this review, “PD-L1 positive” refers to PD-L1 expression at or above the threshold specified by the primary study, most commonly assessed by immunohistochemistry (IHC) using tumor proportion scoring (percentage of viable tumor cells with membranous staining), often with a ≥ 1% cutoff (and less commonly ≥ 5% or ≥ 10%); some studies used scoring approaches that incorporate PD-L1-positive immune cells (e.g., immune-cell scoring or combined scoring). “TMB-high” refers to the study-defined threshold for elevated somatic mutational load, calculated from whole-exome sequencing or targeted next-generation sequencing panels and reported either as mutations per megabase (mut/MB) or as missense single-nucleotide variants per exome. Thresholds varied across reports: while a ≥ 10 mut/Mb cutoff is commonly used in broader oncology, replication-repair deficient/constitutional mismatch repair deficiency cohorts typically fall in the hypermutant to ultrahypermutant range (often tens to hundreds of mut/Mb; some studies used exome-based thresholds such as > ~ 100 missense SNVs/exome). Because assays, panel sizes, and scoring conventions differ, PD-L1 positivity and TMB-high status are not directly comparable across studies and are interpreted descriptively.

### Appraisal of methodological quality

A formal domain-based risk-of-bias tool (e.g., ROBINS-I, QUIPS) was not applied because the available evidence consisted primarily of single arm early exploratory trials, retrospective cohorts, case series, and descriptive biomarker studies with heterogeneous endpoints and analytic frameworks. These study types thus lack structural features required for consistent scoring across domains, and applying a comparative risk tool to largely non comparative designs can produce ratings that are difficult to interpret. Instead, study limitations were qualitatively appraised within each results section and summarized in the discussion. Recurrent risks included small sample sizes, lack of comparator groups, open-label outcome assessment, and confounding by indication. This approach allowed us to evaluate methodological constraints in context and to highlight patterns that were more informative for interpreting this specific body of evidence.

## Results

### Study selection and characteristics

**Fig. 1:**
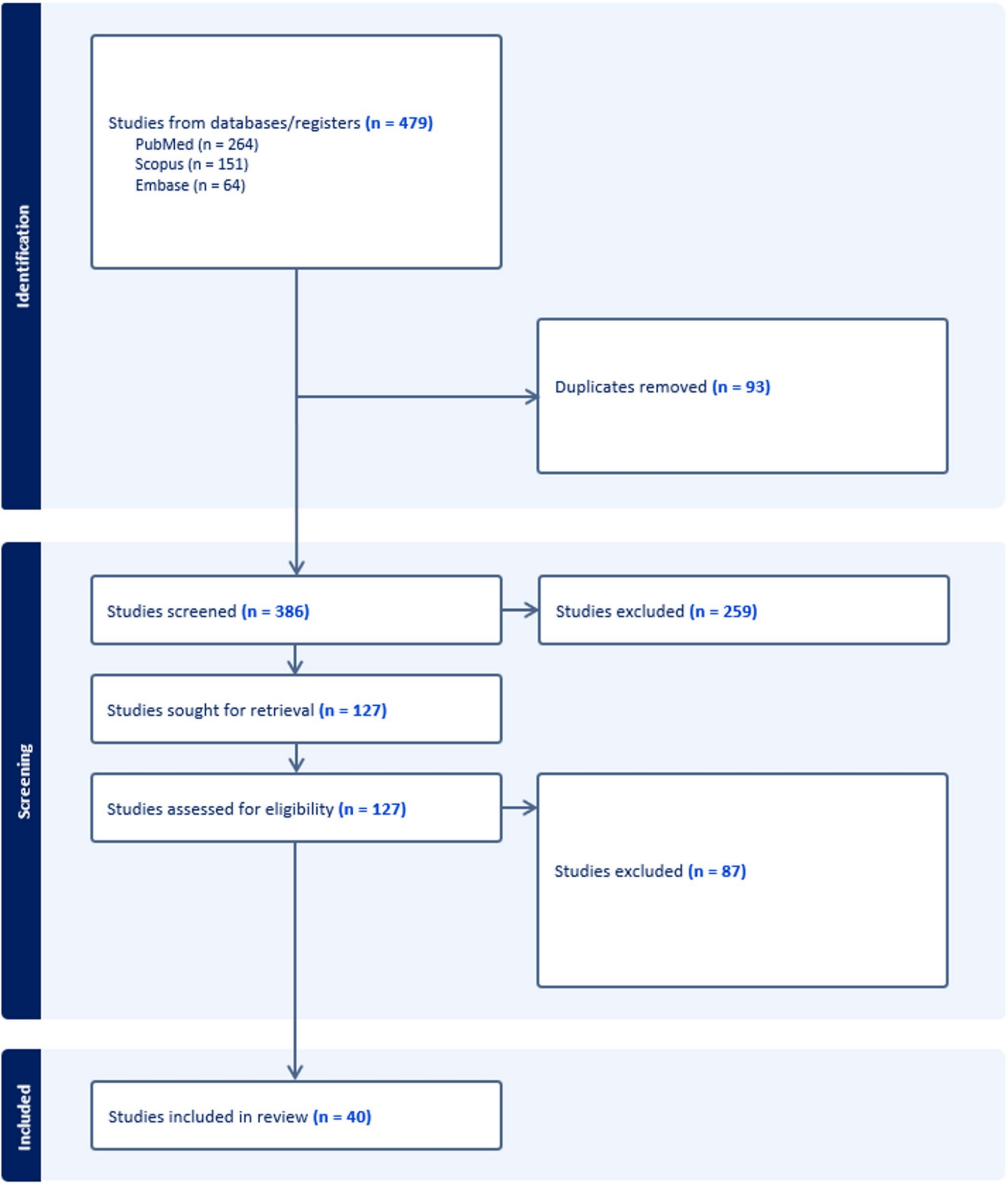
PRISMA flow diagram of the included studies

The PRISMA flow is detailed above (Fig. 1). Studies included multi-cohort prospective trials, basket cohorts, pediatric dose-finding/expansion, single-institution series, case reports, and biomarker/translational works spanning DMG/DIPG, HGG/GBM, MB, EPN, CNS0GCT, and selected non-malignant sellar entities.

### Efficacy in unselected populations: prospective trials

Across prospective studies in unselected pediatric CNS populations, objective responses to single-agent or dual-agent checkpoint inhibitors were uncommon and median progression-free survival (PFS) was short. CheckMate-908 tested nivolumab alone or with ipilimumab (combined group) across multiple newly diagnosed and recurrent cohorts. The diffuse intrinsic pontine glioma (DIPG) group showed median overall survival (OS) 11.7 months (nivolumab) vs. 10.8 months (combined), with > = 3 treatment-related adverse events (TRAEs) in 14.1% and 27.2% of patients respectively [3]. For recurrent/progressive HGG, the median PFS was 1.7 months (nivolumab) vs. 1.3 months (combination). For medulloblastoma, the median PFS was 1.4 months (nivolumab) vs. 2.8 months (combination). For relapsed/resistant ependymoma, the median PFS was 1.4 months (nivolumab) vs. 4.6 months (combination) [3]. There was no OS advantage in any cohort, and prespecified exploratory analyses did not demonstrate PD-L1 as prognostic in this setting [3]. A consolidated view of prospective and basket studies is provided in Table 1. Notably, cross-study comparisons of PFS and disease control should be interpreted cautiously because cohorts differed in treatment line and prior/concomitant modalities (e.g., radiotherapy, systemic chemotherapy, and corticosteroid exposure), which can influence imaging and time-to-event endpoints.

**Table 1:** Prospective & basket studies of checkpoint inhibitors that include pediatric CNS cohorts

Study	Design / Population	Histology/Cohort	Regimen	*N* (evaluable)	Objective Response Rate (ORR)	Disease Control Rate (DCR)	Median Progression Free Survival (PFS)	Median Overall Survival (OS)	Grade ≥ 3 Adverse Events (AEs)	Key notes
Dunkel, 2023	Multi-cohort phase 1/2	Newly diagnosed DIPG; recurrent HGG/MB/EPN	Nivolumab 3 mg/kg q2 w ± Ipilimumab 1 mg/kg q3 w (4 doses, then NIVO 3 mg/kg q2w)	Cohort-specific (Total *N* = 166)	Low (> 20% where reported) across cohorts	NA	HGG 1.7 vs. 1.3 mo; MB 1.4 vs. 2.8 mo; EPN 1.4 vs. 4.6 mo (nivo vs. combo)	DIPG 11.7 vs. 10.8 mo (nivo vs. combo)	14.1% vs. 27.2% (nivo vs. combo); no TR deaths	No survival advantage vs. historical; PD-L1 not prognostic; only 4 HGG had CD8⁺ > 1%
Pappo, 2025	Basket (KEYNOTE-051) pediatric MSI-H subset	7 total; 6 CNS (GBM 4, AA 1, HGG 1)	Pembrolizumab 2 mg/kg (maximum 200 mg) every 3 weeks, up to 35 doses or until discontinuation	7	0% (95% CI 0–41)	14% (95% CI 0–58)	1.7 mo (95% CI 0.4–NR)	7.7 mo (95% CI 1.9–NR)	TRAE 3/7; G3–4 1/7; no fatal AEs	One participant with GBM had a complete response at cycle 6 after initial progression, maintained through cycle 20
Das, 2023	Prospective pilot, hypermutant/RRD	CNS + non-CNS hypermutant	Nivolumab 3 mg/kg IV every 2 weeks, continued until confirmed progression, unacceptable toxicity, or maximum 24 months	20 screened, 10 included in response cohort	20% initial; iBOR 50% (delayed)	20% (95% CI, 6–69)	~ 3.6 mo (overall)	2-year OS (KM): overall ≈ 50% (95% CI 27% − 93%); malignant glioma ≈ 43%	generally tolerable; most were grade ≤ 2. Notable immune-related events included grade 3 pancreatitis with grade 4 lipase in one patient	Frequent pseudoprogression observed under immune-adapted continuation; confirmed responders were enriched among ultra-hypermutant/MSI-indel–high tumors.
André, 2024	Phase 1 pediatric safety run-in	Mixed (includes CNS)	Nivolumab 3 mg/kg IV q2w + 1 of 3 metronomic chemotherapy schedules	*N* = 16 (Arm A *n* = 3; Arm B *n* = 6; Arm C *n* = 7)	0% confirmed	37.5% (6/16 SD)	6-mo PFS 12%	6-mo OS 44% (95% CI 2–33)	≥ G3 AEs frequent in early cycles (8/16 patients); no dose-limiting toxicities (DLT)	Notable individual outcomes: one ATRT patient in arm C remained progression-free at last follow-up after 24 cycles (follow-up 28.5 months); one anaplastic xanthoastrocytoma had PFS ~ 9.3 months and OS 24.5 months.; randomized expansion planned
Davis, 2020	Phase 1/2 pediatric	Non-CNS predominant	Nivolumab 3 mg/kg q2 w	~ 85 (safety) Neuroblastoma *n* = 10	No confirmed ORR in solid tumors	NA	NA	NA	TRAE ≥ G3 ~ 36%; no DLT in confirmation; no AE deaths	Suggests that Nivolumab monotherapy is generally ineffective as a broad treatment for pediatric brain tumors
Loeb, 2022	Phase 1 pediatric	Mainly non-CNS	Avelumab IV Q2W at 10 mg/kg (*n* = 6) or 20 mg/kg (*n* = 15)	Total *N*=21 CNS tumor *N* = 8 (all in 20 mg/kg cohort)	0%	27% in 20 mg/kg cohort	7.7 weeks	NA	Mostly low-grade; limited ≥ G3	Acceptable tolerability; no consistent CNS efficacy signal

### Efficacy in unselected populations: institutional/retrospective

Single-institution experiences corroborate the modest activity signal in biomarker-unselected CNS disease. In a retrospective series by Gikandi et al. from 2014 to 2022, 50 pediatric/young adult patients (median age 14.5 years, 40% CNS) was treated off-label with anti-PD-1/PD-L1 and the overall disease control rate (DCR) was 42 (1 complete response, 2 partial responses, 18 stable disease) [16]. The median PFS of the group was 2.1 months (95% CI 1.4–4.1). The median OS of the group was 9.7 months (95% CI 4.1–18.9) [16]. Institutional activity signals and outcome distributions are summarized in Table 2.

**Table 2:** Retrospective / single-center pediatric CNS ICI experiences

Study	Design / Population	Histology	Regimen	*N*	ORR	DCR	Median PFS	Median OS	Safety / Notable findings
Gikandi, 2024	Retrospective, off-label anti-PD-(L)1 (≈ 40% CNS)	Mixed (solid-tumor, neuro-oncology, hematologic)	Anti-PD-1/PD-L1	50 (*N* = 20 Neurooncology)	6% (1 CR in melanoma, 2 PR in high-grade glioma and CNS germ cell tumor)	42% (incl. 18 SD)	2.1 mo (95% CI 1.4–4.1)	9.7 mo (95% CI 4.1–18.9)	irAEs 44%; ≥ G3 ~ 20%; irAEs associated with better PFS/OS (aHR 0.35; 0.33; *p* = 0.002)
Gorsi, 2019	Single-center nivolumab	HGG, embryonal, EPN, others	Nivolumab IV 3 mg/kg every 2 week	10	3 PR (≈ 15.8%)	N/A	5.5 weeks (range 1.6–24 weeks)	Not reported, 9/10 patients died by data cut-off	Modest activity; acceptable tolerance; adverse events all described as short-lived, mostly grade 2
Tinka, 2025	Retrospective single-institution case series	Ependymoma (6 posterior fossa and 2 ZFTA-fusion positive)	Nivolumab ± DC vaccine (often maintenance)	8	N/A	Sustained CR ≥ 3 y in ZFTA⁺ subset	20 months (range 3–65)	81.5 months	Maintenance PD-1 supported durable CRs in select ZFTA⁺ cases; survival figures are cohort-level, not attributable to ICI alone

In an earlier single-center report of nivolumab for relapsed pediatric CNS tumors (*n* = 19; HGG, EPN, MB, others), responses were rarely recorded (three partial responses), though stable disease occurred in several patients. PD-L1–positive tumors exhibited a trend toward longer OS (13.7 weeks vs. 4.2 weeks unadjusted *p* = 0.08), suggesting a weak enrichment signal but falling short of statistical significance [4]. Taken together, across trial and clinic-based cohorts without biomarker selection, the ORR was generally ≤ 6%, median PFS 1–3 months, and OS < 1 year in DIPG/HGG, consistent with refractory natural history and highlighting a need for biological selection and combination strategies [3, 4, 16].

Beyond monotherapy, early pediatric combination regimens that attempt immune ‘priming’ have reported acceptable tolerability. In a European safety run-in (*N* = 16) testing three metronomic platforms with nivolumab, grade ≥ 3 adverse events were catalogued without unexpected signals; six-month disease control metrics were reported as exploratory and the trial proceeded to expansion [15]. These data support combination-first strategies for otherwise ‘cold’ pediatric CNS tumors [15].

### Efficacy in biomarker-enriched populations

ICI demonstrates greatest efficacy thus far in germline mismatch-repair deficiency (MMRD) and broader replication-repair–deficient (RRD) states. In a prospective pediatric hypermutation pilot of nivolumab enriched for RRD cases, responses often emerged late and pseudoprogression was common; the protocol permitted treatment beyond initial radiographic progression when clinically appropriate, helping mitigate premature discontinuation (*n* = 2 discontinued prematurely) [9]. Two-year overall survival approached ~ 50%; responses were enriched in ultra-hypermutant and MSI/indel-rich tumors [9].

A genomics-first international cohort similarly found that in children with germline replication-repair deficiency, checkpoint blockade led to clinically meaningful and sometimes durable responses, with survival benefits concentrated in proofreading defects due to DNA polymerase epsilon (POLE) or DNA polymerase delta 1 (POLD1) and bMMRD [8]. While study designs preclude a single pooled hazard ratio across disparate tumor types, improved time-to-event outcomes in the hypermutant subsets were consistent, and 6 complete responses (9 partial responses) were documented [8].

By contrast, in a KEYNOTE-051 pediatric cohort restricted to MSI-H solid tumors (*n* = 7; six CNS), pembrolizumab produced ORR 0% (95% CI 0–41) and DCR 14% (95% CI 0–58), with median PFS 1.7 months (95% CI 0.4–NR) and median OS 7.7 months (95% CI 1.9–NR) [17]. Notably, one glioblastoma patient achieved a complete response after initial progression (an iRECIST-like delayed response), emphasizing that isolated durable benefit can occur even when early ORR is negligible [17].

### Histology-specific biomarkers

#### Diffuse midline glioma (DMG)

Clinicopathologic profiling shows variable PD-L1 across DMG subsets and sparse CD3⁺/CD8⁺ infiltration overall, supporting an immunologically “cold” baseline with limited spontaneous priming [18]. Although robust cohort-wide p-values are rarely significant, the directionality, (low TIL density and intermittent PD-L1) aligns with the limited unselected ICI activity described above [18].

#### Pediatric glioblastoma

In a multi-omics analysis, MMR alterations were more frequent than in adults and identifiable by routine MMR immunohistochemistry, nominating candidates for RRD-directed immunotherapy. Correlative analyses linked checkpoint expression patterns to molecular drivers (e.g., p53/TP53) but did not show broad, histology-wide PD-L1 overexpression [19]. In pGBM, PD-L1 expression is detectable and associates with certain lineage/stress markers: PD-L1 correlated positively with OLIG2 (*r* = 0.61; *p* < 0.01) and p53 (*r* = 0.57; *p* < 0.05), with no correlation to Ki-67 (*r* = 0.38; *p* > 0.05), pointing to biology distinct from adult GBM [20].

Transcriptomic profiling of 495 pediatric gliomas identified three immune subtypes, immune-hot (IS-I), immune-altered (IS-II), and immune-cold (IS-III), with macrophages as the dominant infiltrate followed by T cells [21]. Immune-hot tumors displayed higher checkpoint gene expression and the best overall survival [21]. Kaplan–Meier analyses showed significant survival separation among subtypes in both the CBTTC training cohort (median OS 29.8 vs. 19.2 vs. 14.5 years for IS-I vs. IS-II vs. IS-III; *p* < 0.01) and the ICGC validation cohort (> 18 vs. 13.3 vs. 1.99 years; *p* < 0.01) [21].

#### Medulloblastoma

Multiple series report low PD-L1 at baseline, with occasional focal positivity and induction by IFN-γ or radiation in vitro, findings that argue for combination approaches to create an inflamed microenvironment [22]. Quantitatively, most tumors showed ≤ 1% PD-L1-positive cells, and subgroup differences favored higher inducibility in SHH models while baseline expression tended to be lowest in Group 3/4 [22]. In vivo correlative studies show limited survival association for PD-L1 alone: in an independent MB cohort, no PD-L1 expression was detected by validated clones, and survival was instead associated with cytotoxic effectors, e.g., granzyme B + CTL density (*p* = 0.015) and SerpinB1 negativity (trend *p* = 0.050) [12]. A second large expression series reported that B7-H3 (CD276), rather than PD-L1, was frequently expressed in MB, particularly Group 4 (*p* = 0.02 for higher frequency vs. other subgroups), and associated with reduced CD3⁺ infiltration (*p* = 0.041) and fewer γδ T cells (*p* = 0.002) [23 ]. Collectively, these data suggest that alternate checkpoints (e.g., CD276) and myeloid programs may be more dominant than PD-L1 in MB [12, 22, 23].

#### Ependymoma

In relapsed pediatric ependymoma, contemporary single-center and consortial experiences emphasize individualized salvage, including vaccines and checkpoint inhibitors within multimodal plans; durable, unequivocal ICI responses remain uncommon but biologically guided selections are increasingly used [24].

Across pediatric malignant brain tumors. In a population-based Korean series spanning MB, EPN, HGG, ATRT and others, PD-1/PD-L1 expression patterns were not associated with survival after histology stratification. Reported log-rank p-values for OS comparisons by PD-L1 status were *p* = 0.91 (AT/RT), *p* = 0.98 (EPN), and *p* = 0.97 (HGG), underscoring the weak prognostic value of static PD-L1 immunohistochemistry in pediatrics [25]. Isolated case-level activity highlights potential synergy with pathway inhibitors: a child with diffuse leptomeningeal disease experienced a sustained response when nivolumab was paired with a MEK inhibitor after focal radiotherapy, illustrating a rational MAPK-immunotherapy combination in the pediatric CNS [26].

#### CNS germ-cell tumors

In a 50-case series of pediatric CNS germ cell tumors, PD-L1 was positive in 79% of germinomas and ~ 57% of NGGCTs (tumor-cell cutoff > 1%), while PD-1-positive lymphocytes were present in 96% of germinomas and ~ 86% of NGGCTs; choriocarcinoma components showed the most diffuse PD-L1 staining [27].

#### Pituitary adenoma and papillary craniopharyngioma

Although not malignant, pediatric and adolescent pituitary adenomas demonstrate frequent PD-L1 staining with accompanying CD8⁺ T-cell infiltration, highlighting that immune checkpoints are variably expressed even in non-malignant sellar lesions [28]. More broadly, checkpoint expression patterns vary across pediatric CNS histologies. Compared with adults, pediatric papillary craniopharyngiomas show significantly lower PD-L1 expression (*p* < 0.01) and a distinct myeloid/innate signature (higher CD38 [*p* < 0.01], S100A8/A9, MPO [*p* < 0.01]), suggesting different microenvironmental constraints on ICI efficacy in children [29].

### Safety of ICIs in pediatric neuro-oncology

The CheckMate-908 program recorded grade ≥ 3 TRAEs in 14.1% (nivolumab) and 27.2% (nivolumab + ipilimumab) with no new safety signals for pediatrics [3]. Real-world data demonstrated a 44% irAE rate (grade ≥ 3 in 20%) with no treatment-related deaths, and irAEs tracked with favorable outcomes as above [16]. In the pediatric MSI-H cohort of KEYNOTE-051, treatment-related AEs occurred in 3/7 participants; grade 3–4 events in 1/7 (lymphocyte count decreased, pyrexia), with no fatal AEs [17]. Outside the CNS setting, pediatric phase 1–2 data confirm the general tolerability of nivolumab (most common events anemia and fatigue), with no dose-limiting toxicities during dose confirmation and a 3 mg/kg q2w RP2D, context that informs combination designs in neuro-oncology [30]. Cross-study safety and irAE patterns are shown in Table 3.

**Table 3:** Safety summary across pediatric checkpoint experiences

Study	Regimen	Any AE / irAE	Grade ≥ 3	Treatment-related deaths	Key safety notes
Dunkel, 2023	Nivolumab 3 mg/kg IV q2w ± Ipilimumab 1 mg/kg IV 3 weeks	Nivo: 57.6% Combined: 64.2%	14.1% (nivo) vs. 27.2% (combo)	None	No new pediatric safety signals
Gikandi, 2024	Anti-PD-(L)1 (various)	irAEs 44%	~ 20%	None	irAEs correlated with better PFS/OS
Pappo, 2025	Pembrolizumab 2 mg/kg q3w	TRAE 3/7	1/7	None	Delayed CR in GBM despite low ORR
Davis, 2020	Nivolumab 3 mg/kg q2 weeks	Common (anemia, fatigue)	~ 36% ≥ G3	None	7 patients (10%) were discontinued due to AE
André, 2024	Nivolumab 3 mg/kg q2 + Arm A: vinblastine + cyclophosphamide Arm B: capecitabine Arm C: vinblastine + cyclophosphamide + capecitabine	8/16 patients	≥ G3 AEs frequent in early cycles; no DLT	None	No immune-related severe AEs reported
Loeb, 2022	Avelumab 10 mg/kg IV q2 (*n* = 6) 20 mg/kg IV q2 (*n* = 15)	16/21 patients	Limited ≥ G3	None	8 patients (53%) in the 20 mg/kg discontinued due to AEs
Sun, 2018	(Study used to provide context)	–	–	–	Steroids blunt T-cell immunity, favor steroid-sparing where safe

### Prospective/ongoing studies

INFORM2 NivEnt enrolls children/adolescents with relapsed/refractory high-risk solid and CNS tumors in a seamless design (phase I 3 + 3 dose-escalation; phase II basket), uses nivolumab 3 mg/kg q2 weeks with weekly entinostat (2 or 4 mg/m²), and applies Bayesian early stopping for futility; the planned maximum sample is 128 [31]. The design operationalizes biomarker selection (TMB > ~ 100 missense SNVs/exome; PD-L1 mRNA high; MYC/MYCN amplification) and a one-week epigenetic ‘priming’ period before checkpoint blockade, reflecting a mechanistic rationale for HDAC-PD-1 synergy in pediatric tumors [31].

## Discussion

The clinical picture that emerges from the described studies illustrates that PD-1/PD-L1 or CTLA-4 blockade has delivered limited benefit in unselected pediatric CNS tumors, with safety profiles that are generally manageable but efficacy that rarely surpasses historical benchmarks. CheckMate-908, the largest study of anti-PD-1 +/- anti-CTLA-4 monotherapy in this population, showed no survival advantage in any of its five cohorts, including newly diagnosed DIPG and recurrent high-grade glioma, medulloblastoma, and ependymoma [3]. Median OS in newly diagnosed DIPG hovered near historical expectations (~ 11–12 months), and PFS was short across recurrent cohorts despite acceptable tolerability. Exploratory correlative work further highlighted the lack of cytotoxic T-cell presence in these tumors (only 4 HGGs had CD8 + expression > 1%), reinforcing their “cold” phenotype. This data justifies the field’s current pivot away from empiric checkpoint monotherapy and toward biologically enriched enrollment and rational combinations [3].

### Barriers to single-agent checkpoint blockades

Several complementary features of pediatric CNS tumor biology make them challenging targets for ICI therapies. As shown by systematic immunophenotyping of fresh pediatric brain tumor specimens, low PD-1/PD-L1 expression on infiltrating lymphocytes, restricted T-cell clonal expansion, and no consistent correlation between immune infiltrates, grade, mutational load, or survival indicate limited immune contributions to antitumoral immune responses [32]. Supporting this, a study described medulloblastoma as behaving like an “immune desert,” with very low PD-L1 and T-cell gene signatures [11]. Together, this data supports that many pediatric tumors lack both the antigenic landscape and the effector cell support necessary for checkpoin t therapy to succeed without priming [11, 32]. The new pediatric combination therapy with nivolumab layered onto metronomic chemotherapy (e.g., cyclophosphamide/vinblastine-based, capecitabine-based, or multi-agent based) complements the negative monotherapy/prospective CNS trials by showing feasibility of immune-priming backbones [15]. Parallel biomarker work continues to explain histology-linked differences: PD-L1 and T-cell patterns vary between pediatric gliomas, pituitary lesions, and craniopharyngioma, and diverge from adults [20, 28, 29]. These data, together with robust RRD evidence, support a precision approach that first enriches for hypermutation/signature-positive tumors and uses mechanism-matched combinations in otherwise immunologically ‘cold’ disease [14].

Further supporting this is data suggesting that patterns are not uniform across histologies. In DMG, PD-L1 staining tends to be patchy: in one clinicopathologic series of pediatric H3K27M tumors, only a small subset showed convincing PD-L1 labeling and most specimens had sparse CD3⁺/CD8⁺ infiltrates [18]. Pediatric glioblastoma tells a different story. Mismatch-repair defects appear more often than in adult GBM, and simple MMR immunohistochemistry can flag candidates for replication-/repair-deficient biology, and with it, potential sensitivity to immunotherapy [19]. By contrast, medulloblastoma is largely PD-L1–negative across molecular subgroups. Immune escape there seems to lean on non-PD-1 axes and myeloid-skewed programs, while B7-H3 (CD276), often alongside CD24, emerges as a recurrent, targetable surface antigen in pediatric embryonal tumors [11, 13].

### Interpreting responses in hypermutant/RRD disease

By contrast, there is converging evidence that replication-/mismatch-repair–deficient (RRD/dMMR) pediatric cancers, including a sizeable proportion of CNS tumors, are immunologically distinct and can derive meaningful, durable benefit from PD-1 blockade when properly identified. At the biologic level, constitutional mismatch repair deficiency (CMMRD) produces ultra-hypermutation and microsatellite indel signatures that increase neoantigenicity and, in some cases, inflame the tumor microenvironment; gene-level differences matter, with germline MLH1/MSH2 variants conferring earlier, more aggressive phenotypes than PMS2/MSH6 [33].,[34]. Clinically, a prospective pediatric trial that restricted enrollment to tumors with increased TMB and/or MMRD reported a best overall response of 50% with nivolumab, including durable complete responses in malignant glioma, and a 2-year OS of ~ 50%, with delayed immune responses and pseudoprogression featuring prominently [9]. Case-based and early cohort experiences further support activity of anti-PD-1 in CMMRD-associated gliomas, while also highlighting the need for steroid-sparing management and immune response-adapted imaging [35]. Taken together, these data argue for routine MMR IHC and molecular testing in pediatric HGG, at diagnosis and at recurrence, to detect RRD biology upfront [9, 19, 33–35].

While no universally accepted standard exists, published pediatric RRD/CMMRD experiences and contemporary trial paradigms commonly integrate PD-1 blockade early in the course of definitive therapy [9, 36]. Following maximal safe resection, focal radiotherapy is delivered, with steroid minimization whenever feasible, and anti-PD-1 therapy is frequently initiated either during radiotherapy or shortly after its completion, then continued as maintenance until disease progression or unacceptable toxicity [37]. Because delayed responses and radiographic pseudoprogression are well described in hypermutant gliomas treated with PD-1 blockade, immune-adapted response criteria (iRANO/iRECIST) and allowance for treatment beyond initial radiographic progression in clinically stable patients are often incorporated [38].

The KEYNOTE-051 experience underscores an important nuance: even within “immunogenic” banners like MSI-H, pediatric CNS tumors may not behave like adult gastrointestinal cancers. In the pediatric MSI-H cohort (*n* = 7; six CNS primaries), the ORR was 0% by RECIST, and DCR 14%; but notably, a child with glioblastoma achieved a delayed complete response after initial progression, sustaining remission with continued pembrolizumab [17]. This pattern of late tumor regressions after immune-adapted continuation mirrors the delayed responses seen in the hypermutant nivolumab pilot. It also emphasizes that iRANO-concordant trial designs and clinical discipline (e.g., continuing beyond first iUPD when safe) are essential in pediatric neuro-oncology immunotherapy [9, 17].

Outside biomarker-enriched settings, institutional series confirm the picture of tolerable safety with modest activity. In a single-center retrospective review, 10 children with recurrent brain tumors treated with nivolumab had three transient partial responses (mostly at the primary site with progression elsewhere), and longer survivals were observed in PD-L1–positive or higher TMB tumors [4]. A larger single-center “off-label” audit across pediatric solid and CNS tumors found a disease control rate of ~ 37% but low objective responses; immune-related adverse events occurred in roughly half of patients and, as in adults, sometimes co-trended with clinical benefit [16]. Phase 1 pediatric dose-escalation work with avelumab also demonstrated feasibility and a safety profile dominated by low-grade events, but no consistent signals of efficacy in CNS tumors [39]. This shows that checkpoint inhibitors can be safely delivered to children, including those with prior cranial irradiation, but the therapeutic index is narrow unless the underlying tumor biology is favorable [4, 16, 39].

### What constitutes a credible path forward?

#### Routine, early identification of hypermutation and replication-repair deficiency

Clinically, CMMRD imposes a staggering cancer burden in childhood, with CNS tumors as the most frequent neoplasms and gene-dependent differences in onset and survival [33]. Given that pediatric GBM harbors MMR alterations more often than adult GBM, and that routine MMR IHC can rapidly triage candidates, front-line diagnostics should include MMR IHC and, when feasible, tumor-normal sequencing with TMB/MSI and mutational-signature analysis [19, 33]. When RRD biology is present, PD-1 blockade can yield durable disease control, including in malignant gliomas; however, trial designs must anticipate delayed responses and pseudoprogression [9, 17, 35].

#### Imaging phenomena and delayed benefit

Across hypermutant/RRD cohorts, investigators emphasized immune-related response patterns, including pseudoprogression and late conversions to response, necessitating iRECIST-aligned assessment. The pediatric hypermutation pilot reported frequent pseudoprogression and delayed best responses under continued therapy,[9]. while the KEYNOTE-051 pediatric MSI-H cohort documented a complete response arising after initial progression in GBM [17]. These patterns, although not suited to standard early-ORR endpoints, were clinically durable when achieved [9, 17]. Family-based reports from CMMRD cohorts also document prolonged benefit with anti-PD-1 across multiple tumor types, reinforcing the biologic sensitivity of RRD syndromes to checkpoint blockade [40]. In practice, molecularly guided single-patient experiences also reinforce multidisciplinary interpretation of imaging and biomarkers when responses are delayed or atypical [41].

### Turning “cold” tumors “hot” with immune priming combinations

Radiation, oncolytic virotherapy, metronomic chemotherapy, and vaccines can increase antigen release, augment antigen presentation, or shift myeloid/lymphoid balance toward antitumor immunity. For example, pediatric oncolytic HSV-1 therapy (G207) induces a brisk CD8⁺ infiltrate but simultaneously upregulates PD-1/PD-L1, CTLA-4, and IDO within the tumor microenvironment, providing a mechanistic basis for combining virotherapy with checkpoint blockade [42]. Similarly, a multimodal regimen incorporating Newcastle disease virus, hyperthermia, and dendritic-cell vaccination in children with DIPG was feasible and safe in a real-world cohort; while non-randomized, median OS (~ 14.4 months when delivered up front) compared favorably with historical series, supporting further study of immunogenic priming followed by or combined with ICIs [43]. Peptide-based vaccine work in pediatric low-grade glioma demonstrates that robust T-cell activation signatures in blood track with deeper and more durable radiographic responses, suggesting that immunomonitoring can help identify responders and optimize schedules [44]. These priming approaches should be built into next-generation, biomarker-selected trials that incorporate steroid-sparing policies, standardized iRANO triggers, and correlative immune profiling.

### Targeting alternative, developmentally relevant immune checkpoints and antigens

The pediatric tumor immune landscape differs from adults; in medulloblastoma, canonical PD-1/PD-L1 signaling is often absent, whereas myeloid programs and non-PD-1 checkpoints can be dominant, and B7-H3 is commonly expressed [11]. Such findings rationalize trials of B7-H3–directed antibodies or CAR-T cells, LAG-3/TIGIT blockade, and macrophage-reprogramming agents, potentially in combination with ICIs to address lineage-specific immune evasion [11].

### Special situations: germ cell tumors and pituitary tumors

CNS germ cell tumors display frequent PD-L1 expression and inflammatory infiltrates in some series, and scattered pediatric cases with disease control on checkpoint inhibitors have been reported; nonetheless, robust CNS-specific prospective data are lacking and responses appear inconsistent [16]. In pituitary tumors, rare in pediatrics, PD-L1 and CD8⁺ infiltration have been documented, raising hypotheses for selected use in refractory cases, but practice-changing evidence is not yet available [23].

### Safety, neuro-toxicity, and supportive care

Across pediatric studies and real-world series, ICIs have an acceptable safety profile, but immune-related toxicities, including endocrinopathies and, less commonly, neurotoxicity, require vigilance, multidisciplinary management, and clear algorithms to avoid premature discontinuation in the setting of possible pseudoprogression [5, 16, 39]. Steroid-sparing strategies are critical given their negative impact on T-cell function and potential to blunt ICI efficacy; CheckMate-908 allowed low-dose steroids but still observed poor T-cell infiltration and minimal clinical benefit [3, 5].

### Imaging/biopsy pitfalls and procedure-related considerations

In hypermutant gliomas on PD-1 blockade, delayed responses and pseudoprogression are common, mandating immune-adapted criteria (iRANO) and multidisciplinary interpretation before declaring failure [9]. Moreover, neurosurgical and interventional innovations need careful integration with immunotherapy; for example, a case series described aggressive along-tract tumor propagation shortly after laser interstitial thermal therapy (LITT) in heavily pretreated high-grade pediatric tumors (including one treated with ICI), highlighting the need to understand how ablation, radiation, and checkpoint blockade remodel the local microenvironment and white-matter boundaries [45].

### Limitations of current evidence

This review relied on heterogenous non-randomized designs and descriptive studies; consequently, we did not apply a formal, tool-based risk-of-bias score across all reports. Instead methodological limitations were appraised narratively by evidence type. Most interventional studies were single-arm, open-label, and frequently conducted in heavily pretreated populations, making them vulnerable to confounding by indication and selection bias. Retrospective series had similar constraints from incomplete baseline characterization and unmeasured covariates. These features limit causal inference and preclude meta-analysis.

Clinical endpoints and their measurement varied widely across studies. Response criteria ranged from RECIST/iRECIST to RANO/iRANO, and scan timing or central review was seldom standardized. Immune response kinetics such as pseudoprogression and late conversions, were often not captured, which can lower apparent early ORR and confound PFS. Reporting of concomitant and prior therapies was uneven; in particular, corticosteroids, cranial irradiation, and temozolomide can alter T-cell function, mutational/indel burden, and imaging appearances. These factors can confound comparisons of PFS and duration of disease control across studies and limit attribution of outcomes to ICI alone. Finally, small cohorts, short or inconsistent follow up, and early stopping further limit the precision of survival estimates.

Biomarkers also lacked standardization. PD-L1 assays (clones, scoring compartments, cutoffs), sampling timepoints (pre- vs. on-treatment). And dynamic induction by IFN-γ/radiation varied across studies. Genomic measures also differed: TMB “high” thresholds depended on platform/panel size; MSI/MMR was determined by IHC, PCR, or NGS with variable concordance and signature calling was not uniform. WHO classification changes (e.g., DMG H3K27-altered) were not consistently applied in older series, raising the possibility of histologic/molecular misclassification. Publication and language restrictions (english only) and the search cutoff introduce additional reporting bias. Collectively, these factors argue for cautious interpretation of apparent activity in unselected cohorts and for confirmation of biomarker-linked signals in biomarker-enriched, prospectively designed pediatric trials with harmonized diagnostics, iRANO-concordant assessment, steroid-minimization protocols, and integrated correlative profiling.

## Future trials

For high-grade glioma/DMG, we endorse biomarker-driven enrollment that enriches for RRD/hypermutation (confirmed by loss of MMR proteins and/or high TMB with MSI/indel signatures) and builds in immune priming (e.g., hypofractionated radiation, oncolytic virotherapy, or vaccination) before or with PD-1 blockade; these trials should prespecify iRANO-compliant continuation rules and steroid-minimization protocols [3, 9, 42–44]. For medulloblastoma and other PD-L1–low embryonal tumors, combinations that target non-PD-1 checkpoints, myeloid axes, or B7-H3 may be higher-yield than PD-1 monotherapy [11]. Finally, systematic MMR testing should become standard in pediatric HGG at diagnosis and relapse, with rapid IHC screening to triage comprehensive profiling and expedite immunotherapy when indicated [19, 33].

## Conclusion

In sum, the weight of the evidence suggests that immune checkpoint inhibitors are unlikely to change outcomes for most unselected pediatric CNS tumors; however, in the biologically defined context of replication/repair deficiency and hypermutation, durable benefit, including in malignant glioma, is now documented. The path forward is therefore not abandon-but-refine: diagnose RRD early and comprehensively, prime the tumor–immune ecosystem, select combinations that match each histology’s immune contexture, and adopt imaging and toxicity frameworks that respect the kinetics of anti-tumor immunity in the brain.

## Supplementary Information

Below is the link to the electronic supplementary material.

Supplementary Material 1

## Data Availability

This systematic review used previously published data and generated an extracted dataset. All data underpinning the results are presented in the tables within the manuscript. Additional extracted data that were collected during the review but not reported in the article are available from the corresponding author upon reasonable request.
