# Supplementary material for: Should checkpoint inhibitors be reserved for biomarker-selected pediatric brain tumors?
Source: J Neurooncol. 2026 May 16;178(1):2. doi: 10.1007/s11060-026-05605-4 (PMC13179885; doi:10.1007/s11060-026-05605-4)
Supplement: Supplementary file 1 — Supplementary Material 1 [file 11060_2026_5605_MOESM1_ESM.docx]

Supplemental table 1. Search terms

| **Database** | Full Search Syntax | Number of Search Results |
| --- | --- | --- |
| PubMed | ( "Brain Neoplasms"[Mesh] OR "Glioma"[Mesh] OR "Medulloblastoma"[Mesh] OR "Ependymoma"[Mesh] OR brain tumor*[tiab] OR brain neoplasm*[tiab] OR (central nervous system[tiab] OR CNS[tiab]) AND (tumor*[tiab] OR tumour*[tiab] OR neoplasm*[tiab]) OR glioma*[tiab] OR "high-grade glioma"[tiab] OR HGG[tiab] OR glioblastoma[tiab] OR GBM[tiab] OR "diffuse intrinsic pontine glioma"[tiab] OR DIPG[tiab] OR "diffuse midline glioma"[tiab] OR DMG[tiab] OR "brainstem glioma"[tiab] OR medulloblastoma*[tiab] OR ependymoma*[tiab] OR pineoblastoma*[tiab] OR "atypical teratoid rhabdoid"[tiab] OR ATRT[tiab] OR "embryonal tumor*"[tiab] OR ETMR[tiab] OR craniopharyngioma*[tiab] ) AND ( "Immune Checkpoint Inhibitors"[Mesh] OR "Programmed Cell Death 1 Receptor"[Mesh] OR "Programmed Cell Death 1 Ligand 1 Protein"[Mesh] OR "Cytotoxic T-Lymphocyte Antigen 4"[Mesh] OR "immune checkpoint*"[tiab] OR "checkpoint blockade"[tiab] OR ("PD-1"[tiab] OR PD1[tiab] OR "programmed death 1"[tiab]) OR ("PD-L1"[tiab] OR PDL1[tiab] OR CD274[tiab] OR "B7-H1"[tiab] OR B7H1[tiab]) OR ("CTLA-4"[tiab] OR CTLA4[tiab] OR CD152[tiab]) OR ipilimumab[tiab] OR nivolumab[tiab] OR pembrolizumab[tiab] OR cemiplimab[tiab] OR tislelizumab[tiab] OR toripalimab[tiab] OR sintilimab[tiab] OR atezolizumab[tiab] OR durvalumab[tiab] OR avelumab[tiab] OR tremelimumab[tiab] OR pidilizumab[tiab] OR MDV9300[tiab] OR spartalizumab[tiab] ) AND ( "Pediatrics"[Mesh] OR "Child"[Mesh] OR "Adolescent"[Mesh] OR pediatric*[tiab] OR paediatric*[tiab] OR child*[tiab] OR infant*[tiab] OR neonat*[tiab] OR adolescen*[tiab] OR teen*[tiab] OR youth*[tiab] ) | 264 |
| Embase | ('diffuse intrinsic pontine glioma' OR dipg OR 'diffuse midline glioma' OR dmg OR glioblastoma OR gbm OR 'high grade glioma' OR hgg OR medulloblastoma OR ependymoma) AND (ipilimumab OR nivolumab OR pembrolizumab OR atezolizumab OR durvalumab OR avelumab) AND ('immune checkpoint inhibitor'/exp OR 'programmed cell death 1 receptor'/exp OR 'programmed cell death 1 ligand 1'/exp OR 'cytotoxic t lymphocyte antigen 4'/exp) AND ([child]/lim OR [adolescent]/lim) AND [english]/lim NOT ([animals]/lim NOT [humans]/lim) | 64 |
| Scopus | ("brain tumor" OR "brain tumour" OR "brain neoplasm" OR "central nervous system" OR CNS OR tumor OR tumour OR neoplasm OR glioma OR high grade glioma OR hgg OR glioblastoma OR gbm OR "diffuse intrinsic pontine glioma" OR dipg OR "diffuse midline glioma" OR dmg OR "brainstem glioma" OR medulloblastoma OR ependymoma OR pineoblastoma OR atrt OR "atypical teratoid rhabdoid" OR etmr OR craniopharyngioma) AND ( "immune checkpoint*" OR "checkpoint blockade" OR "pd-1" OR pd1 OR "pd-l1" OR pdl1 OR ctla-4 OR ipilimumab OR nivolumab OR pembrolizumab OR atezolizumab OR durvalumab OR avelumab OR tremelimumab OR pidilizumab OR spartalizumab ) AND ( pediatric* OR paediatric* OR child* OR adolescen* OR teen* OR youth* ) | 151 |
